# Secondary malignancies in non‐Hodgkin lymphoma survivors: 40 years of follow‐up assessed by treatment modality

**DOI:** 10.1002/cam4.5139

**Published:** 2022-08-17

**Authors:** Matthew W. Parsons, Calvin Rock, Jonathan J. Chipman, Harsh R. Shah, Boyu Hu, Deborah M. Stephens, Randa Tao, Jonathan D. Tward, David K. Gaffney

**Affiliations:** ^1^ Department of Radiation Oncology Huntsman Cancer Institute, University of Utah Salt Lake City Utah USA; ^2^ Cancer Biostatistics Huntsman Cancer Institute, University of Utah Salt Lake City Utah USA; ^3^ Division of Biostatistics, Department of Population Health Sciences University of Utah Salt Lake City Utah USA; ^4^ Division of Hematology/Hematologic Malignancies Huntsman Cancer Institute, University of Utah Salt Lake City Utah USA

**Keywords:** adverse effects, chemotherapy, non‐Hodgkin lymphoma, radiation, secondary malignancy

## Abstract

**Background:**

Survivors of non‐Hodgkin lymphoma (NHL) have increased secondary malignancy (SM) risk. We quantified this risk by patient and treatment factors.

**Methods:**

Standardized incidence ratios (SIR, observed‐to‐expected [O/E] ratio) were assessed in 142,637 NHL patients diagnosed from 1975 to 2016 in the National Cancer Institute's Surveillance, Epidemiology, and End Results Program. Comparisons were made between subgroups in terms of their SIRs relative to respective endemic populations.

**Results:**

In total, 15,979 patients developed SM, more than the endemic rate (O/E 1.29; *p* < 0.05). Compared with white patients, relative to respective endemic populations, ethnic minorities had a higher risk of SM (white O/E 1.27, 95% CI 1.25–1.29; black O/E 1.40, 95% CI 1.31–1.48; other O/E 1.59, 95% CI 1.49–1.70). Relative to respective endemic populations, patients who received radiotherapy had similar SM rates to those who did not (O/E 1.29 each), but irradiated patients had increased breast cancer (*p* < 0.05). Patients who received chemotherapy had higher SM rates than those who did not (O/E 1.33 vs. 1.24, *p* < 0.05) including more leukemia, Kaposi sarcoma, kidney, pancreas, rectal, head and neck, and colon cancers (*p* < 0.05).

**Conclusions:**

This is the largest study to examine SM risk in NHL patients with the longest follow‐up. Treatment with radiotherapy did not increase overall SM risk, while chemotherapy was associated with a higher overall risk. However, certain subsites were associated with a higher risk of SM, and they varied by treatment, age group, race and time since treatment. These findings are helpful for informing screening and long‐term follow‐up in NHL survivors.

## Graphical abstract

Survivors of non‐Hodgkin lymphoma (NHL) have increased secondary malignancy (SM) risk. Treatment with radiotherapy did not increase overall SM risk, while chemotherapy was associated with a higher overall risk. However, certain sub‐sites were associated with higher risk of SM, and they varied by treatment, age group, race and time since treatment." cd_value_code="text

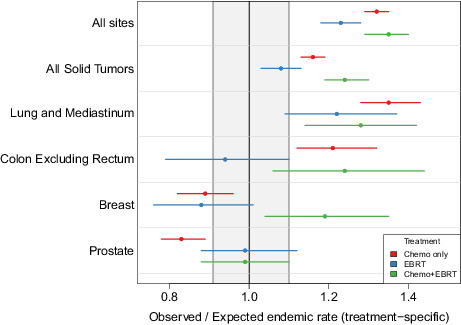

## 1 BACKGROUND

In 2021, an estimated 81,560 patients will be diagnosed with non‐Hodgkin lymphoma (NHL) in the United States, and 20,720 patients will die of the disease.^1^ Both the incidence and long‐term survivorship of NHL have increased over the past 50 years, resulting in an ever‐growing group of survivors.^2^ NHL survivors are at risk of numerous late effects, either intrinsic to the disease itself or secondary to treatments. These include the development of various secondary malignancies (SM).^3^, ^4^ A variety of treatments have been implicated in this risk including chemotherapy,^5^, ^6^, ^7^, ^8^, ^9^, ^10^ stem cell transplant,^11^, ^12^, ^13^, ^14^, ^15^, ^16^ and radiotherapy.^3^, ^4^, ^17^, ^18^ Treatment guidelines for NHL vary with subtype and stage, but chemotherapy and radiation are the predominant treatment modalities employed.^19^

Given the growing survivor pool, an accurate and detailed understanding of patient and treatment characteristics associated with SM risk is essential to inform follow‐up and screening protocols. Our group previously used the National Cancer Institute's Surveillance, Epidemiology, and End Results (SEER) database to assess SM risk in NHL survivors.^3^ Since that publication, SEER has grown and now captures chemotherapy as a treatment modality. Accordingly, we sought to update the SM risk in NHL survivors with 15 additional years of follow‐up and data and include the assessment of SM among patients who receive chemotherapy.

## 2 METHODS

The SEER program collects and publishes cancer incidence and survival data from 18 population‐based cancer registries and three supplemental registries that cover approximately 34.6% of the U.S. population. SEER began collecting data on January 1, 1975 and has information on over 10 million patients with in situ and invasive cancer. The SEER Registries collect data on patient demographics, tumor site, morphology, stage, treatment, and follow‐up for vital status.^20^ The SEER Program statistical analysis software package (SEER*Stat, version 8.3.8)^21^ was used to identify patients diagnosed with any stage of NHL as their first primary malignancy between 1975 and 2016.

The SEER*Stat Multiple Primary‐SIR tool was used to calculate the standard incidence ratio (SIR) and absolute excess risk (AER) for SM by comparing the number of SMs these patients experienced with the number expected based on incidence rates for the U.S. population. These analyses were adjusted for age, gender, race, patient‐years at risk (PYs), and year of NHL diagnosis. Individuals who developed an SM within 2 months of NHL diagnosis or whose NHL diagnosis was not their first primary cancer were excluded from the analysis. Only invasive malignancies were considered as SM, and in situ disease was excluded. Additionally, basal and squamous cell skin cancers were excluded.

The observed PYs for each case were compiled as the time from 2 months after the date of NHL diagnosis to the date of death, date of developing second NHL, last follow‐up, diagnosis of SM, or study close (December 31, 2016), whichever occurred first. Endemic population cancer incidence rates specific for 5‐year age groups, gender, race, and calendar‐year intervals were multiplied by the observed accumulated PYs to obtain the estimated number of cancers expected. The SIR was then expressed as the ratio of observed‐to‐expected (O/E) cases. The AER was determined by subtracting the expected number from the observed number of second cancers and dividing the difference by PYs. AER was expressed per 10,000 PYs.

Risks of SM were stratified by gender, age at NHL diagnosis, time since diagnosis (latency), and treatment (radiotherapy vs. no radiotherapy, chemotherapy vs. no chemotherapy). Confidence intervals (95% CIs) were based on the assumption that the observed number of SMs was distributed as a Poisson variable. SIR and AER are complementary measures of the incidence of an event (SM) in a subpopulation compared with the entire population. Both are based on comparing the observed number of events in the subpopulation with the number of events that would be expected if the risk profile for the subpopulation matched the index population. Because, as an individual age, their risk of an event is altered, the calculation of the expected number of events is adjusted for these variables. In addition, fixed characteristics that affect event rates, such as gender and race, are incorporated into the calculation of the expected number of events.

We compared the risk of various subgroups of interests by patient characteristics, treatment modality, and latency. For each subgroup of interest, risks were standardized to their own age, gender, race, patient‐years at risk (PYs), and year of NHL diagnosis matched endemic population. That is, comparisons between subgroups are relative to different endemic populations and the interpretation is how the subgroups' risk compare to each of their own matched endemic population. Statistical significance was determined on the basis of 95% confidence intervals overlapping or not, which is a conservative assessment of significance when comparing intervals from two different populations.^22^ It should be noted that the length of follow‐up does not affect SIR: The number of observed events increases, but so does the number of expected events and the PYs.

## 3 RESULTS

### 3.1 Patient population

A total of 141,451 patients were included in this analysis. Of note, 64,380 (46%) patients had to follow up beyond 5 years, and 34,742 (25%) had to follow up beyond 10 years. In total, 923,475 patient‐years of follow‐up were recorded. Patient characteristics are shown in Table 1. SM occurred in 15,979 patients (11.3%) with a mean latency of 94 months. Some patients developed more than one SM, and there were a total of 18,151 SMs observed. Characteristics of the patients who developed an SM are shown in Table 2.

**TABLE 1:** Patients with NHL at risk for developing secondary malignancies by receipt of chemotherapy and RT

	All patients	EBRT	No RT	Chemotherapy	No chemotherapy
Total patients	141,451	32,212	107,226	87,318	54,133
Male	76,775 (54.3)	17,432 (54.1)	58,332 (54.4)	49,299(56.4)	27,476 (50.8)
Female	64,676 (45.7)	14,780 (45.9)	48,894 (45.6)	38,019 (43.6)	26,657 (49.2)
Patients with secondary malignancy	15,979	3913	11,846	9133	6846
Age at NHL diagnosis	61.5	59.6	62.1	59.6	64.7
Age at secondary	70.2	69.8	70.5	69.0	71.7
Patient‐years at risk	923,475	239,465	669,206	549,513	373,962
Average months follow‐up	78.4	89.2	74.9	75.5	82.9
Race					
White	120,617 (85.3)	27,480 (85.3)	91,414 (85.3)	74,581 (85.4)	46,036 (85.0)
Black	10,220 (7.2)	1982 (6.2)	8056 (7.5)	6240 (7.1)	3980 (7.4)
Other	9666 (6.8)	2656 (8.2)	6907 (6.4)	6202 (7.1)	3464 (6.4)
Unknown	948 (0.7)	94 (0.3)	849 (0.8)	295 (0.3)	653 (1.2)

Abbreviations: EBRT, external beam radiation therapy; NHL, non‐Hodgkin lymphoma; RT, radiation therapy.

**TABLE 2:** Demographics of patients who developed secondary malignancies

	All patients	EBRT	No RT	Chemotherapy	No chemotherapy
Total patients	15,979	3913	11,846	9133	6846
Male	9293 (58.2)	2242 (57.3)	6945 (58.6)	5409 (59.2)	3884 (56.7)
Female	6686 (41.8)	1671 (42.7)	4901 (41.4)	3724 (40.7)	2962 (43.3)
Age at NHL diagnosis	62.4	60.3	63.1	61.2	64.0
Patient‐years at risk	147,032	41,332	103,475	85,176	61,856
Average months follow‐up	94.0	109.0	89.0	94.6	93.2
Race					
White	14,130 (88.4)	3433 (87.8)	10,490 (88.6)	8099 (88.7)	6031 (88.1)
Black	978 (6.1)	190 (4.9)	773 (6.5)	531 (5.8)	447 (6.5)
Unknown	866 (5.4)	290 (7.4)	569 (4.8)	501 (5.5)	365 (5.3)
Other	5 (<0.1)	0 (0)	5 (<0.1)	2(<0.1)	3(<0.1)

Abbreviations: EBRT, external beam radiation therapy; NHL, non‐Hodgkin lymphoma; RT, radiation therapy.

### 3.2 Overall SM risk

Overall, SMs occurred at a significantly higher rate in NHL survivors than in the general U.S. population (SIR 1.29, 95% CI 1.27–1.31). Malignancies at significantly increased risk compared to the endemic rate included head and neck, stomach, colon, anal, liver, lung, bone and joint, soft tissue, melanoma, breast cancer in males, bladder, kidney, thyroid, Hodgkin lymphoma, leukemia, and Kaposi sarcoma (Table 3). The SMs with the greatest excess risk were lung cancer and leukemia (AER 6.65 and 5.49, respectively). Interestingly, rectal cancer, breast cancer in females, and prostate cancer had lower risks of occurrence in the study cohort relative to the endemic‐matched U.S. population (Table 3).

**TABLE 3:** Standardized incidence ratios and absolute excess risk for second cancers in patients with non‐hodgkin lymphoma

Site	Patients 142,837		Patient‐years 984,155	
	Observed	Excess risk	O/E	95% CI
All sites	18,151	41.13	1.29^a^	1.27–1.31
All solid tumors	14,126	16.81	1.13^a^	1.11–1.15
Head and neck	493	1.63	1.48^a^	1.35–1.62
Esophagus	178	0.20	1.13	0.97–1.30
Stomach	314	0.62	1.24^a^	1.11–1.38
Colon excluding rectum	1316	1.48	1.12^a^	1.06–1.19
Rectum and rectosigmoid	348	−0.64	0.85^a^	0.76–0.94
Anus, anal canal, and anorectum	77	0.37	1.90^a^	1.50–2.38
Liver, gallbladder, and biliary	346	0.51	1.17^a^	1.05–1.30
Pancreas	387	−0.32	0.92	0.84–1.02
Lung and mediastinum	2760	6.65	1.31^a^	1.26–1.36
Bone and joint	37	0.24	2.76^a^	1.94–3.80
Soft tissue	99	0.27	1.37^a^	1.12–1.67
Melanoma	715	1.64	1.29^a^	1.20–1.39
Breast	1509	−1.32	0.92^a^	0.88–0.97
Female breast	1480	−1.43	0.91^a^	0.87–0.96
Male breast	29	0.11	1.62^a^	1.08–2.32
Gynecologic	604	−0.43	0.93	0.86–1.01
Prostate	2248	−1.98	0.92^a^	0.88‐0.96
Testes	17	−0.02	0.89	0.52–1.43
Penis	15	0.02	1.16	0.65–1.91
Bladder	1010	2.00	1.24^a^	1.17–1.32
Kidney and renal pelvis	596	1.99	1.49^a^	1.37–1.61
Brain	149	0.09	1.06	0.90–1.25
Thyroid	328	1.79	2.16^a^	1.93–2.41
Hodgkin lymphoma	249	2.17	7.02^a^	6.18–7.95
Myeloma	221	0.15	1.07	0.93–1.22
Leukemia	956	5.49	2.30^a^	2.16–2.45
Mesothelioma	52	0.13	1.31	0.98–1.72
Kaposi sarcoma	139	1.26	9.20^a^	7.74–10.87
Miscellaneous	439	1.20	1.37^a^	1.24–1.50

Abbreviations: CI, confidence interval; O/E, observed‐to‐expected.

^a^ *p* < 0.05 observed versus expected relative to endemic population rate.

### 3.3 SM risk by the timing of diagnosis

When evaluating NHL diagnosis before or after December 31, 2001, and relative to their respective endemic populations, SM risk was significantly greater in patients diagnosed in 2002 or later (O/E 1.49, 95% CI 1.45–1.52 vs. O/E 1.19, 95% CI 1.17–1.21). Of note, this increased risk was seen both in those with both B‐cell and T‐cell lymphoma, though the effect size was larger in those with B‐cell lymphoma (B cell O/E 1.19, 95% CI 1.17–1.22 vs. O/E 1.49, 95% CI 1.46–1.53; T‐cell O/E 1.30, 95% 1.23–1.38 vs. O/E 1.53, 95% CI 1.40–1.66). Compared with treatment‐specific endemic populations, this significantly increased risk was present in patients who received no therapy (O/E 1.44, 95% CI 1.38–1.50 vs. 1.13, 95% 1.09–1.17), chemotherapy alone (O/E 1.54, 95% CI 1.49–1.59 vs. 1.21, 95% CI 1.18–1.24), and radiation alone (O/E 1.48, 95% CI 1.36–1.61 vs. 1.16, 95% CI 1.10–1.22). This observation was not seen in patients who received both chemotherapy and radiation (O/E 1.43, 95% CI 1.33–1.53 vs. 1.32, 95% CI 1.25–1.38). Specific cancers with increased risk among patients with a 2002 NHL diagnosis and later include kidney, brain, and thyroid cancers as well as Hodgkin lymphoma, multiple myeloma, and leukemia (Table S1).

### 3.4 SM risk by patient characteristics

We analyzed SM risk according to baseline patient and disease characteristics. Relative to respective endemic populations, no difference in SM risk was observed between males and females (O/E 1.28 vs. 1.29). Compared with endemic populations noted as black and white as per SEER terminology, SM risk was higher in minority patients compared to white patients (white O/E 1.27, 95% CI 1.25–1.29; black O/E 1.40, 95% CI 1.31–1.48; other O/E 1.59, 95% CI 1.49–1.70). Comparisons of baseline disease characteristics did not observe a difference in SM risk over respective endemic populations between B‐cell and T‐cell lymphoma (B‐cell O/E 1.31, 95% CI 1.28–1.33: T‐cell O/E 1.37, 95% CI 1.31–1.44) but were more elevated in patients with advanced stage at diagnosis with Stage III–IV disease (O/E 1.37, 95% CI 1.34–1.39) than in patients with Stage I–II (O/E 1.25, 95% CI 1.23–1.27). With regards to the age at diagnosis, patients were at the greatest risk of SM if NHL diagnosis occurred at age <25 (O/E 3.22, 95% CI 2.71–3.73). Although this risk decreased with increasing age, it remained statistically greater than the endemic rate even in the oldest patients regardless of treatment received (age 25–49, O/E 1.70, 95% CI 1.63–1.76; age 50–74, O/E 1.26, 95% CI 1.24–1.29; age 75+, O/E 1.10, 95% CI 1.06–1.14).

### 3.5 SM risk by treatment modality

Receipt of external beam radiation was not found to alter the overall SM risk compared with unirradiated patients relative to their respective endemic populations (O/E 1.29, 95% CI 1.25–1.33 vs. 1.29, 95% CI 1.27–1.31) (Table 4). However, certain SMs demonstrated differences in incidence between irradiated and unirradiated patients and their respective endemic populations. Patients who received radiation were at significantly increased risk of female breast cancer and decreased risk of leukemia compared with unirradiated patients and each of their respective endemic populations. Although unirradiated women had significantly decreased rates of breast cancer compared with their endemic rate (O/E 0.87, 95% CI 0.82–0.93), the risk in radiated patients was not statistically different from their endemic rate (O/E 1.02 95% CI 0.93–1.12). Although there was not a statistically significant difference in prostate cancer risk between the radiated and unirradiated groups and their respective endemic populations, unirradiated patients had a lower risk of prostate cancer than their endemic population (O/E 0.90, 95%, CI 0.84–0.94), whereas radiated patients were not statistically different from their endemic population (O/E 0.99, 95% CI 0.91–1.07).

**TABLE 4:** Comparison of standardized incidence ratios and absolute excess risk for patients by receipt of radiation therapy

Site	EBRT				No RT			
	Patients 32,440		Patient‐years 255,987		Patients 108,159		Patient‐years 712,491	
	Observed	Excess risk	O/E	95% CI	Observed	Excess risk	O/E	95% CI
All sites	4461	39.01	1.29^a^	1.25–1.33	13,433	42.11	1.29^a^	1.27–1.31
All solid tumors	3555	19.14	1.16^a^	1.12–1.20	10,370	16.11	1.12^a^	1.10–1.15
Head and neck	134	2.00	1.62^a^	1.36–1.92	351	1.48	1.43^a^	1.29–1.59
Esophagus	41	0.10	1.07	0.77–1.45	136	0.26	1.16	0.97–1.37
Stomach	88	0.96	1.39^a^	1.11–1.71	221	0.49	1.19^a^	1.04–1.35
Colon excluding rectum	312	0.88	1.08	0.96–1.20	987	1.73	1.14^a^	1.07–1.22
Rectum and rectosigmoid	86	−0.68	0.83	0.67–1.03	256	−0.63	0.85^a^	0.75–0.96
Anus, anal canal, and anorectum	21	0.44	2.13^a^	1.32–3.26	55	0.35	1.84^a^	1.38–2.39
Liver, gallbladder, and biliary	89	0.65	1.23	0.99–1.51	253	0.47	1.15^a^	1.02–1.31
Pancreas	104	0.11	1.03	0.84–1.24	282	−0.41	0.91	0.80–1.02
Lung and mediastinum	642	5.01	1.25^a^	1.16–1.35	2072	7.16	1.33^a^	1.27–1.39
Bone and joint	17	0.53	5.05^a^	2.94–8.09	20	0.14	2.03^a^	1.24–3.14
Soft tissue	29	0.44	1.64^a^	1.10–2.36	69	0.22	1.29^a^	1.01–1.64
Melanoma	168	1.33	1.25^a^	1.07–1.46	537	1.76	1.30^a^	1.20–1.42
Breast	419	0.44	1.03	0.93–1.13	1061	−2.01	0.88^a^	0.83–0.94
Female breast	412	0.34	1.02^b^	0.93–1.12	1040	−2.12	0.87^a^	0.82–0.93
Male breast	7	0.10	1.62	0.65–3.34	21	0.11	1.57	0.97–2.40
Gynecologic	157	−0.18	0.97	0.82–1.14	437	−0.52	0.92	0.84–1.01
Prostate	591	−0.22	0.99	0.91–1.07	1624	−2.64	0.90^a^	0.85–0.94
Testes	1	−0.17	0.18	0.00–1.02	15	0.02	1.13	0.63–1.86
Penis	4	0.03	1.28	0.34–3.27	11	0.02	1.14	0.57–2.04
Bladder	220	0.92	1.12	0.98–1.28	778	2.42	1.28^a^	1.20–1.38
Kidney and renal relvis	150	2.06	1.54^a^	1.31–1.87	440	2.00	1.48^a^	1.34–1.62
Brain	36	0.04	1.03	0.72–1.43	111	0.11	1.07	0.88–1.29
Thyroid	87	1.89	2.26^a^	1.81–2.79	234	1.73	2.11^a^	1.85–2.40
Hodgkin lymphoma	57	1.87	6.25^a^	4.74–8.10	187	2.26	7.26^a^	6.25–8.37
Myeloma	47	−0.10	0.95	0.70–1.26	171	0.24	1.11	0.95–1.29
Leukemia	184	3.25	1.83^a^ ^,^ ^b^	1.57–2.11	761	6.35	2.46^a^	2.29–2.65
Mesothelioma	20	0.41	2.09^a^	1.28–3.23	32	0.03	1.08	0.74–1.53
Kaposi sarcoma	32	1.09	7.81^a^	5.34–11.03	107	1.35	9.95^a^	8.15–12.02
Miscellaneous	106	1.05	1.34^a^	1.10–1.62	328	1.28	1.39^a^	1.24–1.54

Abbreviations: CI, confidence interval; EBRT, extreme beam radiation therapy; O/E, observed‐to‐expected; RT, radiation therapy.

^a^ *p* < 0.05 observed versus expected relative to respective endemic rate.

^b^ *p* < 0.05 EBRT versus no RT relative to each treatment's respective endemic rate.

Relative to each of their endemic populations, chemotherapy patients were at the increased overall risk of SM compared with those who did not receive chemotherapy (O/E 1.33, 95% CI 1.30–1.35 vs. O/E 1.24, 95% CI 1.21–1.26) (Table 5). This included significantly increased risks of head and neck, colon, rectum, pancreas, and kidney cancers as well as leukemia and Kaposi sarcoma. With regard to leukemia, chemotherapy patients were at increased risk of lymphocytic and nonlymphocytic leukemia (myeloid and monocytic), as well as acute myeloid leukemia but at decreased risk of chronic lymphocytic leukemia (Table S2). Patients treated with chemotherapy were at decreased risk of prostate cancer.

**TABLE 5:** Comparison of standardized incidence ratios and absolute excess risk for patients by receipt of chemotherapy

	Chemotherapy				No chemotherapy			
	Patients 87,939		Patient‐years 583,809		Patients 54,698		Patient‐years 400,346	
Site	Observed	Excess risk	O/E	95% CI	Observed	Excess risk	O/E	95% CI
All sites	10,402	43.96	1.33^a^ ^,^ ^b^	1.30–1.35	7749	37.02	1.24^a^	1.21–1.26
All solid tumors	8191	21.44	1.18^a^ ^,^ ^b^	1.15–1.21	5935	10.07	1.07^a^	1.05–1.10
Head and neck	325	2.33	1.72^a^ ^,^ ^b^	1.54–1.92	168	0.60	1.17	1.00–1.36
Esophagus	111	0.38	1.25^a^	1.03–1.51	67	−0.06	0.96	0.75–1.23
Stomach	177	0.67	1.28^a^	1.10–1.49	137	0.54	1.19	1.00–1.40
Colon excluding rectum	769	2.36	1.22^a^ ^,^ ^b^	1.13–1.31	547	0.19	1.01	0.93–1.10
Rectum and rectosigmoid	224	−0.06	0.98^b^	0.86–1.12	124	−1.47	0.68^a^	0.56–0.81
Anus, anal canal, and anorectum	47	0.42	2.07^a^	1.52–2.76	30	0.31	1.69^a^	1.14–2.41
Liver, gallbladder, and biliary	217	0.89	1.32^a^	1.15–1.50	129	−0.05	0.98	0.82–1.17
Pancreas	238	0.17	1.04	0.92–1.19	149	−1.04	0.78^a^	0.66–0.92
Lung and mediastinum	1558	6.72	1.34^a^	1.27–1.40	1202	6.54	1.28^a^	1.21–1.35
Bone and joint	21	0.23	2.71^a^	1.68–4.14	16	0.26	2.83^a^	1.61–4.59
Soft tissue	61	0.35	1.51^a^	1.16–1.94	38	0.16	1.20	0.85–1.65
Melanoma	419	1.78	1.33^a^	1.20–1.46	296	1.45	1.24^a^	1.11–1.39
Breast	861	−0.65	0.96	0.90–1.02	648	−2.30	0.88^a^	0.81–0.95
Female breast	846	−0.73	0.95	0.89–1.02	634	−2.45	0.87^a^	0.80–0.94
Male breast	15	0.08	1.48	0.83–2.44	14	0.15	1.79	0.98–3.01
Gynecologic	345	−0.16	0.97	0.87–1.08	259	−0.83	0.89	0.78–1.00
Prostate	1203	−3.21	0.87^a^ ^,^ ^b^	0.82–0.92	1045	−0.18	0.99	0.93–1.06
Testes	9	−0.07	0.70	0.32–1.33	8	0.05	1.29	0.56–2.25
Penis	6	−0.02	0.83	0.30–1.81	9	0.08	1.57	0.72–2.98
Bladder	566	2.02	1.26^a^	1.16–1.37	444	1.97	1.22^a^	1.11–1.33
Kidney and renal pelvis	381	2.66	1.69^a^ ^,^ ^b^	1.52–1.86	215	1.01	1.23^a^	1.07–1.41
Brain	76	−0.07	0.95	0.75–1.19	73	0.31	1.21	0.95–1.52
Thyroid	215	2.16	2.41^a^	2.10–2.76	113	1.26	1.80^a^	1.49–2.17
Hodgkin lymphoma	158	2n.35	7.55^a^	6.42–8.83	91	1.91	6.26^a^	5.04–7.69
Myeloma	110	−0.06	0.97	0.80–1.17	111	0.45	1.19	0.98–1.44
Leukemia	612	6.57	2.68^a^ ^,^ ^b^	2.47–2.90	344	3.92	1.84^a^	1.65–2.04
Mesothelioma	31	0.16	1.41	0.96–2.01	21	0.08	1.18^a^	0.73–1.81
Kaposi sarcoma	102	1.59	11.15^a^ ^,^ ^b^	9.08–13.53	37	0.78	6.21^a^	4.37–8.56
Miscellaneous	244	1.25	1.43^a^	1.25–1.62	195	1.14	1.31^a^	1.13–1.50

Abbreviations: CI, confidence interval; O/E, observed‐to‐expected.

^a^ *p* < 0.05 observed versus expected relative to respective endemic rate.

^b^ *p* < 0.05 chemotherapy versus no chemotherapy relative to each treatment's endemic rate.

**FIGURE 1:**
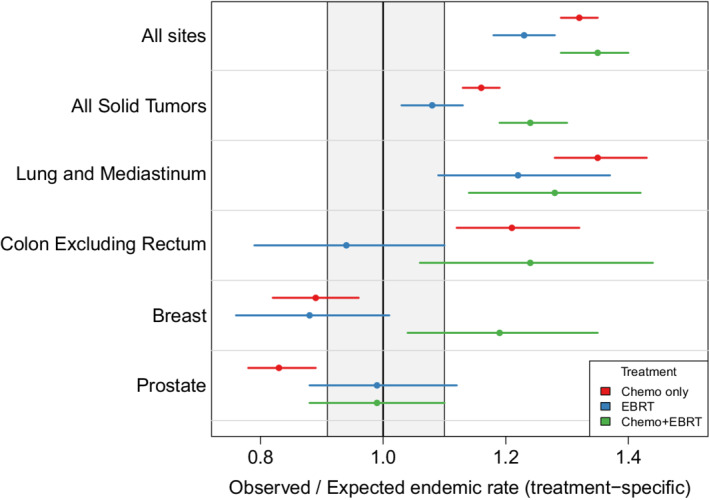
Observed‐to‐expected (O/E) ratio of selected malignancies for which screening is common by treatment modality. The gray box represents the 0.9–1.1 O/E ratio and is included to provide a sense of clinical relevance.

We further stratified patients into four treatment groups (no therapy, radiation alone, chemotherapy alone, and chemotherapy and radiation) in an attempt to isolate the effects of each treatment modality relative to each of their endemic populations (Table S3). The overall risk of SM was significantly elevated in all groups, including those not treated with chemotherapy or radiation. Relative to each of their endemic populations, the overall risk was further elevated in the chemotherapy alone (O/E 1.32, 95% CI 1.29–1.35) and chemotherapy plus radiation (O/E 1.35, 95% CI 1.29–1.40) groups compared to patients who received no therapy (O/E 1.24, 95% CI 1.21–1.27) or radiation alone (O/E 1.23, 95% CI 1.18–1.28). No significant differences in SM risk were observed between the radiation alone and no therapy groups including overall risk and cancer subtypes. Compared to the no therapy group, and relative to respective endemic populations, patients who received chemotherapy alone had an increased risk of head and neck, kidney, and thyroid cancers along with leukemia and Kaposi sarcoma; they had a decreased risk of prostate cancer (Table S3). Compared to the no therapy group, and relative to respective endemic populations, the chemotherapy and radiation group had significantly increased risks of head and neck cancer and female breast cancer. Comparing the chemotherapy alone and chemotherapy plus radiation groups, and relative to respective endemic populations, increased risk of female breast cancer in the chemotherapy plus radiation group was the only statistically significant difference (Table S3). The risk of female breast cancer with chemotherapy alone was less than its endemic rate (O/E 0.88, 95% CI 0.81–0.96). However, treatment with chemotherapy and radiation was associated with a greater risk of female breast cancers as compared to its endemic rate (O/E 1.18, 95% CI 1.03–1.34) and was also significantly greater than any other treatment group's risk relative to their endemic population (Figure 1).

### 3.6 SM risk by latency

**FIGURE 2:**
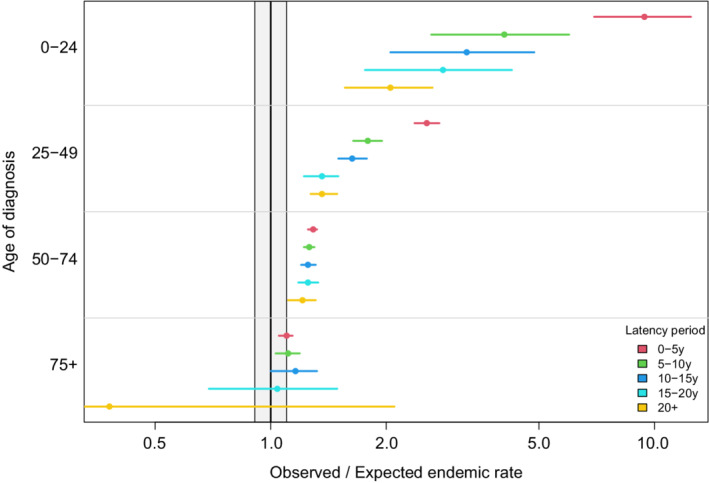
Observed‐to‐expected (O/E) ratio for all secondary malignancies by age and latency from NHL diagnosis displayed on a logarithmic scale. The gray box represents the 0.9–1.1 O/E ratio and is included to provide a sense of clinical relevance. NHL, non‐Hodgkin lymphoma.

There was insufficient evidence to observe an overall difference in risk of SM by years from NHL diagnosis with O/E of 1.28, 1.30, and 1.31 for latencies of 0–10 years, 10–20 years, and >20 years, respectively (Table S4). However, we observed an association between the risk of certain malignancy subtypes and the latency with head and neck, female breast, and bladder cancers being more common at latencies of more than 10 years (Table S4). Conversely, relative to endemic rates, the risks of leukemia, Kaposi sarcoma, thyroid cancer, and kidney cancer were less than 10 years or greater from NHL diagnosis than within the first 10 years (Table S4). The latency period of SM was associated with age at NHL diagnosis. Relative to respective endemic populations, those diagnosed prior to age 50 were associated with shorter latency periods within the first 15 years (Figure 2).

## 4 DISCUSSION

**FIGURE 3:**
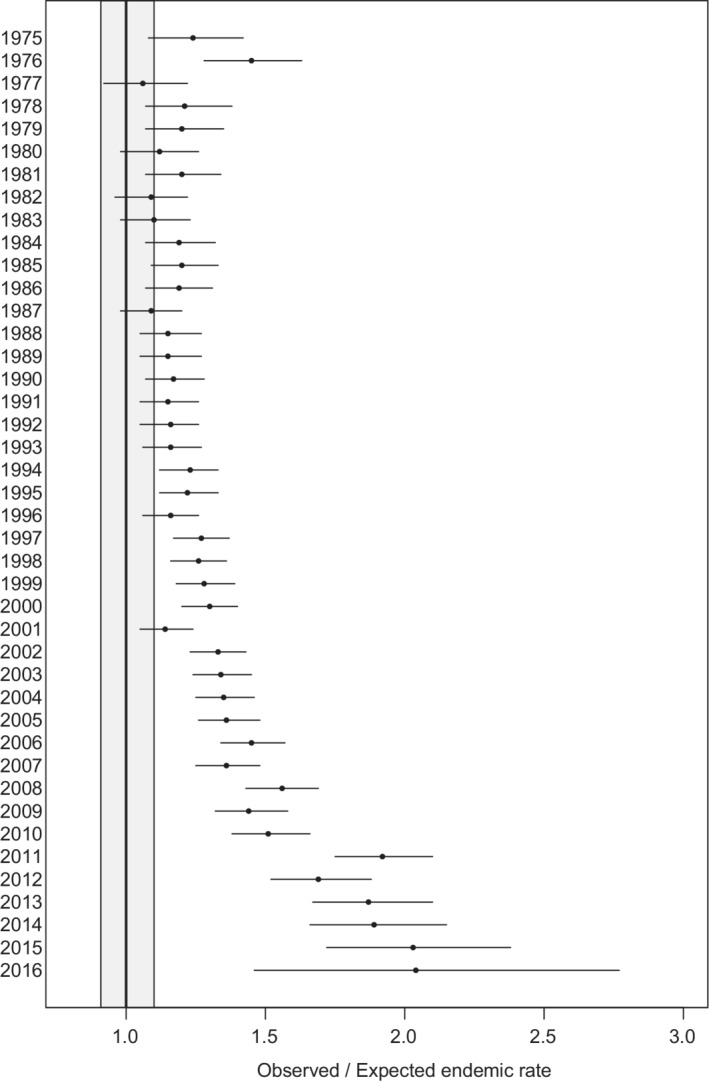
Observed‐to‐expected (O/E) ratio for all secondary malignancies by year of NHL diagnosis. The gray box represents the 0.9–1.1 O/E ratio and is included to provide a sense of clinical relevance. NHL, non‐Hodgkin lymphoma.

Previous studies have observed increased SM risk in NHL.^3^, ^4^, ^11^, ^13^ The results of this study expand upon the 2005 results of Tward et al.^3^ and now represent the largest study of SM in NHL survivors with the longest follow‐up. Importantly, treatment factors recently made available in SEER*Stat allowed us to assess SM in chemotherapy patients which were unavailable in SEER to prior investigators. Our results are consistent with previous reports that the risk of SM is increased in NHL survivors, however, the magnitude of risk is increased in our study compared to the 2005 results (O/E 1.29, 95% CI 1.27–1.31 vs. O/E 1.14, 95% CI 1.12–1.16) (Figure 3). While the risk for specific SM subtypes was largely comparable between our study and the 2005 study, we found novel associations including increased risk of stomach, anal, biliary, and male breast cancers and decreased risk of rectal cancers.

We also stratified patients diagnosed before and after the end of 2001 as the cohort included in Tward et al were diagnosed with NHL prior to 2002. This showed that patients diagnosed after 2001 have a higher risk of SM for all treatment modalities apart from chemoradiation and relative to each era's endemic population. Considering differences in treatment of NHL between the studies, one significant change is the widespread addition of rituximab to chemotherapy regimens for B‐cell lymphomas after 1997.^23^, ^24^ Data on the risk of SM in patients treated with rituximab is mixed with some studies suggesting an increased risk,^16^ and others finding no difference.^25^, ^26^ Additionally, incorporation of R‐EPOCH (rituximab, etoposide, prednisone, vincristine, cyclophosphamide, doxorubicin) into the chemotherapy catalog for the treatment of aggressive B‐cell lymphomas in 2002 could have contributed to increased rates of secondary leukemias due to the addition of etoposide.^27^ Further, temporal differences in the utilization of stem cell transplants and aggressive salvage regimens in the treatment of NHL could be a contributing factor. Importantly, the increased risk of SM in 2002 and beyond was seen in both B‐cell and T‐cell lymphomas, albeit with a larger increase in B‐cell lymphoma, this suggests that while novel B‐cell targeted therapeutics may play some role, they are not solely responsible for the increased risk among patients diagnosed since 2002. Additionally, any potential effect of chemotherapeutics on SM risk would not explain the concurrent increase of SMs observed in the no therapy and radiation alone groups especially since modern radiation trends for the treatment of NHL have been to use smaller fields and lower radiation doses.^28^, ^29^ Therefore, the increased risk since 2002 may be due to different endemic referent populations and factors not captured in SEER including increased cancer screening, increased healthcare utilization, and use of second‐line therapies.

There was not sufficient evidence to observe a gender difference between their endemic‐standardized risk for overall risk for SM, which is consistent with the findings of Tward et al.^3^ This is, however, contrary to the findings of Travis et al who found a higher risk in males than females compared to their respective endemic rates.^4^ In our study, minority patients were at increased SM risk as compared to white patients relative to each of their endemic populations. It should be noted, that this analysis cannot account for all potential confounding variables that may impact SM risk between racial groups including possible genetic, lifestyle, and comorbidity differences between groups. Nevertheless, this finding raises meaningful concerns. The etiology for this inequality cannot be determined through SEER but may relate to previously described healthcare inequalities faced by minority patients including decreased access to healthcare leading to lower rates of cancer screening and decreased detection of in situ disease, decreased follow‐up, and more advanced stage at diagnosis, along with mistrust in the medical system and systemic inequities.^30^, ^31^, ^32^, ^33^, ^34^, ^35^ Our finding of increased SM risk in NHL survivors adds to the already described inequalities faced by non‐white patients and interventional studies to decrease the healthcare gap should be explored in the future.

Consistent with prior studies, we did not observe an overall SM difference between patients who received radiation and patients who did not receive radiation relative to their endemic populations. However, any radiation‐ with or without chemotherapy‐ was associated with a higher risk of female breast cancer, again consistent with the prior study.^3^ We did not observe an increase in endemic rates in female breast cancer among patients treated with radiation or chemotherapy alone, only in those treated with both modalities. This finding is novel and suggests that breast cancer screening may be of added value for women who receive the combination of chemotherapy and radiation as these women had the greatest increase in risk compared to endemic rates. However, in young patients (age <25) radiotherapy alone was observed to have increased female breast cancer rates relative to the referent population (O/E 3.08, 95% CI 1.54–5.52). An important consideration when considering the risk of breast SMs is that SEER does not contain information on NHL location, radiation treatment fields, or dose to the breast which would be expected to have a major impact on SM rates. Since our findings suggest the risk of female breast cancer with radiotherapy in patients diagnosed <25 years old even at short latencies, they support the International Guideline Harmonization Group and American College of Radiology recommendations for breast cancer screening prior to age 40 in patients undergoing radiation.^36^, ^37^ However, our data also show an increased risk of secondary breast cancer in women under the age of 25 who do not undergo radiation (O/E 4.00, 95% CI 2.33–6.40) suggesting these women may also benefit from earlier screening.

We observed chemotherapy patients be at increased risk of SM relative to its endemic population which has not previously been reported. Previous reports from other sources have shown an increased risk of secondary hematologic and bladder cancers.^5^, ^6^, ^8^ Our analysis suggested patients treated with chemotherapy were also at risk for cancers of the head and neck, thyroid, and kidney. Importantly, these risks remained elevated relative to the endemic population when we identified patients treated with chemotherapy alone, without radiotherapy. The large patient population available in SEER likely allowed us to identify these additional associations which could not be seen in smaller studies.

Breast, head and neck and bladder secondary cancers were most common at latencies of over 10 years after NHL diagnosis. This finding is consistent with prior reports which have shown a long latency period between radiation and the development of secondary breast cancers.^38^ Secondary soft tissue sarcomas are also historically highly associated with radiotherapy treatment,^39^ but they were not correlated with a latency period in our data. A number of the SMs that were elevated in chemotherapy relative to its endemic population, including kidney and thyroid cancers, leukemia, and Kaposi sarcoma, were more common at shorter latencies under 10 years. The findings regarding leukemia and Kaposi sarcoma are expected, as the previously established latency for treatment‐related leukemia is 5–7 years, and Kaposi sarcoma is likely related to the immunosuppression seen during chemotherapy.^40^, ^41^

This study is limited by features inherent in a retrospective database analysis. The accuracy of treatment coding in SEER is not perfectly accurate with a reported sensitivity of 68% for receipt of chemotherapy and 80% for radiation.^42^ Additionally, NHL is a heterogenous disease family with numerous subtypes that cannot be fully parsed out in the SEER database. Patients who received no radiation or chemotherapy, for example, may have different disease characteristics which could not be fully appreciated. SEER is unable to account for comorbid conditions or cancer risk factors, such as smoking status, germline genetic mutations, and Human Immuno Virus status, when calculating SIR and AER. Additionally, limited granularity in chemotherapy data limits our ability to isolate certain drugs or even receipt of stem cell transplants, that may be associated with increased SM risk in patients treated with chemotherapy. Similarly, the radiation data is limited and so associations with radiation field or dose are unable to be explored. Due to these limitations, subgroups (such as treatment modalities) are not compared assuming to have controlled for patient characteristics. Rather, each subgroup is standardized relative to its own endemic population which gives insight into SMs for which there may be increased screening value. Finally, it is important to note that we cannot definitively separate true SM from synchronous or metachronous cancers. We excluded cancers that were diagnosed within 2 months of the initial NHL diagnosis as these are unlikely to be true SM. This cut‐off was in keeping with prior studies and allows for a high level of sensitivity for SM, but may still classify as SM some cancers that may be more accurately considered synchronous or metachronous.

In conclusion, this is the largest study to examine SM risk in patients with NHL and has the longest follow‐up. Treatment with radiotherapy did not increase the overall SM risk compared to a matched population, while chemotherapy was associated with a higher overall risk. However, certain sub‐sites were associated with a higher risk of SM, and they varied by treatment modality, age group, race, and time since treatment. While these findings cannot quantify an individual patient's risk of developing a secondary malignancy, they are helpful for informing clinicians on the importance of screening and long‐term follow‐up in NHL survivors.

## AUTHORS CONTRIBUTION

All listed authors made substantial contributions to the conception, design, or data analysis of this study. They were involved in drafting or revising the manuscript. Each author gave final approval of the version to be published and agree to be accountable for all aspects of the work.

## FUNDING INFORMATION

The authors received no funding to support this work.

## CONFLICT OF INTEREST

The authors have no conflict of interest to declare.

## Supporting information


Tables S1–S4

## Data Availability

The data that support the findings of this study are available in the supplementary material of this article. Ethical approval was not needed from an institutional review board.
