# Supplementary material for: Secondary malignancies in non‐Hodgkin lymphoma survivors: 40 years of follow‐up assessed by treatment modality
Source: Cancer Med. 2022 Aug 17;12(3):2624–36. doi: 10.1002/cam4.5139 (PMC9939160; doi:10.1002/cam4.5139)
Supplement: Supplementary file 1 — Tables S1–S4 [file CAM4-12-2624-s001.docx]

Supplemental Table 1: Comparison of Standardized Incidence Ratios and Absolute Excess Risk for Patients by Era of Diagnosis

|  | 1975-2001 | | | | | 2002-2017 | | | | |
| --- | --- | --- | --- | --- | --- | --- | --- | --- | --- | --- |
|  | Patients 75,760 | | Patient Years 614,819 | | | Patients 65,691 | | Patient Years 308,656 | | |
| Site | Observed | Excess Risk | O/E | 95% CI | | Observed | Excess Risk | O/E | 95% CI | |
| All sites | 11,367 | 27.8 | 1.19*# | 1.17 | 1.21 | 6,784 | 68.08 | 1.49* | 1.45 | 1.52 |
| All Solid | 9,344 | 13.51 | 1.11*# | 1.08 | 1.13 | 4,782 | 23.49 | 1.19* | 1.16 | 1.22 |
| Head and Neck | 333 | 1.69 | 1.50* | 1.35 | 1.67 | 160 | 1.49 | 1.44* | 1.22 | 1.68 |
| Esophagus | 117 | 0.2 | 1.12 | 0.93 | 1.35 | 61 | 0.21 | 1.13 | 0.86 | 1.45 |
| Stomach | 216 | 0.56 | 1.21* | 1.05 | 1.38 | 98 | 0.72 | 1.32* | 1.07 | 1.6 |
| Colon Excluding Rectum | 933 | 1.19 | 1.09* | 1.02 | 1.16 | 383 | 2.07 | 1.21* | 1.09 | 1.34 |
| Rectum and Rectosigmoid | 257 | -0.65 | 0.86* | 0.76 | 0.97 | 91 | -0.61 | 0.82 | 0.66 | 1.01 |
| Anus, Anal Canal and Anorectum | 45 | 0.31 | 1.81* | 1.32 | 2.43 | 32 | 0.5 | 2.05* | 1.4 | 2.89 |
| Liver, Gallbladder and Biliary | 201 | 0.34 | 1.12 | 0.97 | 1.29 | 145 | 0.86 | 1.24* | 1.04 | 1.46 |
| Pancreas | 245 | -0.4 | 0.9 | 0.79 | 1.02 | 142 | -0.15 | 0.97 | 0.81 | 1.14 |
| Lung and Mediastinum | 1,829 | 5.85 | 1.27* | 1.21 | 1.33 | 931 | 8.25 | 1.41* | 1.32 | 1.5 |
| Bone and Joint | 31 | 0.34 | 3.52* | 2.39 | 4.99 | 6 | 0.04 | 1.31 | 0.48 | 2.84 |
| Soft Tissue | 66 | 0.31 | 1.45* | 1.12 | 1.85 | 33 | 0.19 | 1.24 | 0.85 | 1.74 |
| Melanoma | 418 | 1.42 | 1.29* | 1.17 | 1.42 | 297 | 2.1 | 1.30* | 1.16 | 1.46 |
| Breast | 1,048 | -1.01 | 0.94* | 0.88 | 1 | 461 | -1.93 | 0.88* | 0.8 | 0.96 |
| Female Breast | 1,025 | -1.19 | 0.93* | 0.87 | 0.99 | 455 | -1.91 | 0.88* | 0.8 | 0.96 |
| Male Breast | 23 | 0.18 | 2.01* | 1.27 | 3.01 | 6 | -0.02 | 0.92 | 0.34 | 2.01 |
| Gynecologic | 410 | -0.52 | 0.92 | 0.84 | 1.02 | 194 | -0.25 | 0.96 | 0.83 | 1.1 |
| Prostate | 1,535 | -2.45 | 0.90* | 0.86 | 0.95 | 713 | -1.01 | 0.96 | 0.89 | 1.03 |
| Testes | 10 | -0.04 | 0.78 | 0.38 | 1.44 | 7 | 0.02 | 1.11 | 0.44 | 2.29 |
| Penis | 13 | 0.07 | 1.55 | 0.83 | 2.66 | 2 | -0.08 | 0.44 | 0.05 | 1.57 |
| Bladder | 696 | 2.41 | 1.30* | 1.2 | 1.4 | 314 | 1.16 | 1.14* | 1.01 | 1.27 |
| Kidney and Renal Pelvis | 344 | 1.44 | 1.38* | 1.24 | 1.53 | 252 | 3.09 | 1.67* | 1.47 | 1.89 |
| Brain | 79 | -0.23 | 0.84# | 0.67 | 1.05 | 70 | 0.73 | 1.51* | 1.18 | 1.91 |
| Thyroid | 115 | 0.45 | 1.35*# | 1.11 | 1.62 | 213 | 4.5 | 3.20* | 2.79 | 3.66 |
| Hodgkin Lymphoma | 144 | 1.82 | 6.01*# | 5.06 | 7.07 | 105 | 2.87 | 9.15* | 7.49 | 11.08 |
| Myeloma | 112 | -0.31 | 0.85# | 0.7 | 1.02 | 109 | 1.07 | 1.47* | 1.21 | 1.77 |
| Leukemia | 517 | 3.71 | 1.89*# | 1.73 | 2.07 | 439 | 9.09 | 3.07* | 2.79 | 3.38 |
| Mesothelioma | 36 | 0.14 | 1.35 | 0.95 | 1.87 | 16 | 0.09 | 1.23 | 0.7 | 2 |
| Kaposi Sarcoma | 118 | 1.61 | 9.83* | 8.14 | 11.78 | 21 | 0.55 | 6.76* | 4.18 | 10.34 |
| Miscellaneous | 310 | 1.17 | 1.33* | 1.19 | 1.49 | 129 | 1.28 | 1.48* | 1.23 | 1.76 |

*P<0.05 Observed vs. Expected relative to era-specific endemic population rate

# P<0.05 1975-2001 vs 2002-2017 and relative to each era-specific endemic population rate

Supplemental Table 2: Incidence of Sub-types of Leukemia Stratified by Receipt of Chemotherapy

|  | Chemotherapy | | | |  | No Chemotherapy | | | |  |
| --- | --- | --- | --- | --- | --- | --- | --- | --- | --- | --- |
|  | Patients 87,939 | | Patient Years 583,809 | | | Patients 54,698 | | Patient Years 400,346 | | |
| Disease | Observed | Excess Risk | O/E | 95%CI | | Observed | Excess Risk | O/E | 95% CI | |
| All Leukemia | 612 | 6.57 | 2.68*# | 2.47 | 2.9 | 344 | 3.92 | 1.84* | 1.65 | 2.04 |
| Acute Lymphocytic Leukemia | 26 | 0.33 | 3.86* | 2.52 | 5.66 | 7 | 0.05 | 1.44 | 0.58 | 2.97 |
| Chronic Lymphocytic Leukemia | 70 | -0.55 | 0.68*# | 0.53 | 0.87 | 119 | 0.88 | 1.42* | 1.18 | 1.7 |
| Other Lymphocytic Leukmia | 11 | 0.06 | 1.48 | 0.74 | 2.66 | 16 | 0.26 | 2.79* | 1.59 | 4.53 |
| Acute Non-Lymphocytic Leukemia | 430 | 6.12 | 5.91*# | 5.36 | 6.49 | 148 | 2.2 | 2.47* | 2.09 | 2.9 |
| Acute Myeloid Leukemia | 381 | 5.45 | 6.05*# | 5.46 | 6.69 | 128 | 1.92 | 2.50* | 2.08 | 2.97 |
| Acute Monocytic Leukemia | 25 | 0.37 | 7.23* | 4.68 | 10.68 | 9 | 0.15 | 3.21* | 1.46 | 6.09 |
| Chronic Myeloid Leukemia | 56 | 0.46 | 1.91* | 1.44 | 2.48 | 38 | 0.35 | 1.59* | 1.12 | 2.18 |
| Other Myeloid/Monocytic Leukemia | 10 | 0.12 | 3.14* | 1.5 | 5.78 | 5 | 0.05 | 1.79 | 0.58 | 4.17 |
| Other Acute Leukemia | 24 | 0.3 | 3.73* | 2.39 | 5.55 | 11 | 0.13 | 1.91 | 0.95 | 3.42 |
| Aleukemic, Subleukemic and NOS | 9 | 0.04 | 1.31 | 0.6 | 2.49 | 11 | 0.12 | 1.76 | 0.88 | 3.16 |

*P<0.05 Observed vs. Expected relative to treatment-specific endemic population rate

# P<0.05 Chemotherapy vs No Chemotherapy and relative to each treatment-specific endemic population rate

Supplemental Table 3: Comparison of Standardized Incidence Ratios and Absolute Excess Risk for Patients by Treatment Group

|  | No Chemotherapy or Radiation | | | | Chemotherapy Alone | | | | Radiation Alone | | | | Chemotherapy and Radiation | | | |
| --- | --- | --- | --- | --- | --- | --- | --- | --- | --- | --- | --- | --- | --- | --- | --- | --- |
|  | Patients 39,734 | | Patient Years 279,835 | | Patients 68,425 | | Patient Years 432,656 | | Patients 14,101 | | Patient Years 113,852 | | Patients 18,339 | | Patient Years 142,135 | |
| Site | Observed | Excess Risk | O/E | 95% CI | Observed | Excess Risk | O/E | 95% CI | Observed | Excess Risk | O/E | 95% CI | Observed | Excess Risk | O/E | 95% CI |
| All sites | 5,499 | 38.03 | 1.24* | 1.21-1.27 | 7,934 | 44.75 | 1.32*# | 1.29-1.35 | 2,132 | 34.81 | 1.23* | 1.18-1.28 | 2,329 | 42.37 | 1.35*# | 1.29-1.40 |
| All Solid Tumors | 4,194 | 10.09 | 1.07* | 1.04-1.11 | 6,176 | 20.00 | 1.16*# | 1.13-1.19 | 1,653 | 10.36 | 1.08* | 1.03-1.13 | 1,902 | 26.17 | 1.24*# | 1.19-1.30 |
| Head and Neck | 110 | 0.32 | 1.09 | 0.89-1.31 | 241 | 2.24 | 1.67*# | 1.47-1.90 | 56 | 1.36 | 1.38* | 1.04-1.79 | 78 | 2.51 | 1.85*# | 1.46-2.30 |
| Esophagus | 53 | 0.12 | 1.07 | 0.80-1.40 | 83 | 0.35 | 1.22 | 0.97-1.52 | 14 | -0.43 | 0.74 | 0.41-1.25 | 27 | 0.52 | 1.38 | 0.91-2.01 |
| Stomach | 92 | 0.42 | 1.15 | 0.92-1.40 | 129 | 0.53 | 1.22* | 1.02-1.45 | 43 | 0.86 | 1.29 | 0.94-1.74 | 45 | 1.04 | 1.49* | 1.09-1.99 |
| Colon Excluding Rectum | 395 | 0.69 | 1.05 | 0.95-1.16 | 592 | 2.41 | 1.21* | 1.12-1.32 | 145 | -0.86 | 0.94 | 0.79-1.10 | 167 | 2.27 | 1.24* | 1.06-1.44 |
| Rectum and Rectosigmoid | 91 | -1.27 | 0.72* | 0.58-0.88 | 165 | -0.22 | 0.95 | 0.81-1.10 | 32 | -1.90 | 0.60* | 0.41-0.84 | 54 | 0.30 | 1.08 | 0.81-1.42 |
| Anus, Anal Canal and Anorectum | 19 | 0.22 | 1.49 | 0.90-2.33 | 36 | 0.43 | 2.09* | 1.46-2.89 | 10 | 0.46 | 2.10* | 1.10-3.87 | 11 | 0.42 | 2.16* | 1.08-3.87 |
| Liver, Gallbladder and Biliary | 90 | -0.14 | 0.96 | 0.77-1.18 | 163 | 0.87 | 1.30* | 1.11-1.52 | 37 | 0.16 | 1.05 | 0.74-1.45 | 52 | 1.04 | 1.40* | 1.04-1.83 |
| Pancreas | 102 | -1.21 | 0.78* | 0.61-0.91 | 180 | 0.11 | 1.03 | 0.88-1.19 | 46 | -0.51 | 0.89 | 0.65-1.18 | 58 | 0.60 | 1.17 | 0.89-1.52 |
| Lung and Mediastinum | 858 | 6.89 | 1.29* | 1.21-1.38 | 1,214 | 7.34 | 1.35* | 1.28-1.43 | 319 | 5.13 | 1.22* | 1.09-1.37 | 323 | 4.92 | 1.28* | 1.14-1.42 |
| Bone and Joint | 9 | 0.18 | 2.25* | 1.03-4.27 | 11 | 0.12 | 1.88 | 0.94-3.37 | 7 | 0.48 | 4.46* | 1.79-9.19 | 10 | 0.58 | 5.57* | 2.67-10.42 |
| Soft Tissue | 23 | 0.01 | 1.01 | 0.64-1.52 | 46 | 0.36 | 1.50* | 1.10-2.00 | 14 | 0.49 | 1.66 | 0.90-2.78 | 15 | 0.41 | 1.63 | 0.91-2.70 |
| Melanoma | 213 | 1.42 | 1.23* | 1.07-1.41 | 324 | 1.90 | 1.36* | 1.22-1.52 | 80 | 1.62 | 1.30* | 1.03-1.62 | 88 | 1.09 | 1.21 | 0.97-1.50 |
| Breast | 452 | -2.33 | 0.87* | 0.80-0.96 | 609 | -1.80 | 0.89* | 0.82-0.96 | 184 | -2.26 | 0.88 | 0.76-1.01 | 235 | 2.61 | 1.19*# | 1.04-1.35 |
| Female Breast | 441 | -2.53 | 0.86* | 0.78-0.95 | 599 | -1.85 | 0.88* | 0.81-0.96 | 181 | -2.34 | 0.87 | 0.75-1.01 | 231 | 2.48 | 1.18*# | 1.03-1.34 |
| Male Breast | 11 | 0.19 | 1.95 | 0.97-3.49 | 10 | 0.05 | 1.29 | 0.62-2.38 | 3 | 0.08 | 1.46 | 0.29-4.26 | 4 | 0.12 | 1.77 | 0.49-4.52 |
| Gynecologic | 179 | -0.85 | 0.88 | 0.76-1.02 | 258 | -0.31 | 0.95 | 0.84-1.07 | 74 | -0.89 | 0.88 | 0.69-1.10 | 83 | 0.39 | 1.07 | 0.85-1.33 |
| Prostate | 741 | -0.28 | 0.99 | 0.92-1.06 | 883 | -4.16 | 0.83*# | 0.78-0.89 | 287 | -0.15 | 0.99 | 0.88-1.12 | 304 | -0.29 | 0.99 | 0.88-1.10 |
| Testes | 7 | 0.10 | 1.64 | 0.66-3.38 | 8 | -0.02 | 0.89 | 0.38-1.75 | 0 | -0.16 | 0,00 | 0.00-2.04 | 1 | -0.19 | 0.27 | 0.00-1.53 |
| Penis | 8 | 0.14 | 1.94 | 0.84-3.82 | 3 | -0.06 | 0.54 | 0.11-1.59 | 1 | -0.05 | 0.65 | 0.01-3.63 | 3 | 0.10 | 1.87 | 0.38-5.47 |
| Bladder | 329 | 2.40 | 1.26* | 1.12-1.40 | 449 | 2.43 | 1.31* | 1.19-1.43 | 113 | 1.30 | 1.15 | 0.95-1.38 | 107 | 0.62 | 1.09 | 0.89-1.32 |
| Kidney and Renal Pelvis | 148 | 0.81 | 1.18 | 1.00-1.39 | 292 | 2.77 | 1.70*# | 1.51-1.90 | 66 | 1.71 | 1.42* | 1.10-1.81 | 84 | 2.34 | 1.66* | 1.32-2.05 |
| Brain | 58 | 0.55 | 1.36* | 1.03-1.76 | 53 | -0.18 | 0.87 | 0.65-1.14 | 14 | -0.26 | 0.83 | 0.45-1.39 | 22 | 0.28 | 1.22 | 0.76-1.85 |
| Thyroid | 74 | 1.03 | 1.64* | 1.29-2.06 | 160 | 2.18 | 2.43*# | 2.07-2.84 | 36 | 1.71 | 2.17* | 1.52-3.01 | 51 | 2.04 | 2.32* | 1.73-3.06 |
| Hodgkin Lymphoma | 66 | 1.99 | 6.47* | 5.01-8.24 | 121 | 2.44 | 7.77* | 6.44-9.28 | 24 | 1.75 | 5.86* | 3.76-8.73 | 33 | 1.97 | 6.57* | 4.52-9.23 |
| Myeloma | 85 | 0.65 | 1.27* | 1.02-1.57 | 86 | -0.03 | 0.99 | 0.79-1.22 | 23 | -0.16 | 0.93 | 0.59-1.39 | 24 | -0.05 | 0.97 | 0.62-1.45 |
| Leukemia | 265 | 4.70 | 1.98* | 1.75-2.24 | 496 | 7.41 | 2.83*# | 2.59-3.09 | 75 | 2.13 | 1.48* | 1.16-1.85 | 109 | 4.15 | 2.18* | 1.79-2.63 |
| Mesothelioma | 13 | 0.01 | 1.02 | 0.54-1.74 | 19 | 0.05 | 1.13 | 0.68-1.76 | 8 | 0.28 | 1.68 | 0.72-3.31 | 12 | 0.51 | 2.50* | 1.29-4.36 |
| Kaposi Sarcoma | 21 | 0.61 | 5.23* | 3.23-7.99 | 86 | 1.83 | 12.77*# | 10.21-15.77 | 16 | 1.25 | 8.81* | 5.03-14.30 | 16 | 0.97 | 7.02* | 4.01-11.41 |
| Miscellaneous | 125 | 0.75 | 1.20 | 1.00-1.43 | 203 | 1.63 | 1.53* | 1.33-1.76 | 66 | 2.03 | 1.54* | 1.19-1.96 | 40 | 0.27 | 1.10 | 0.79-1.50 |

*P<0.05 Observed vs. Expected relative to treatment-specific endemic population rate

#P<0.05 Compared to No chemotherapy or Radiation and relative to each treatment-specific endemic population rate

Supplemental Table 4: Comparison of Absolute Excess Risk and Standardized Incidence Ratios for Selected Malignancies by Latency from NHL Diagnosis

|  | 2-119 months | | 120-239 months | | 240+ months | |
| --- | --- | --- | --- | --- | --- | --- |
|  | Persons 142,637 | PY 706,422 | Persons 37,831 | PY 214,677 | Persons 10,310 | PY 63,056 |
| Site | Excess Risk | O/E | Excess Risk | O/E | Excess Risk | O/E |
| All sites | 39.56 | 1.28* | 44.51 | 1.30* | 47.29 | 1.31* |
| All Solid Tumors | 14.56 | 1.12* | 20.35 | 1.15* | 30.01 | 1.22*# |
| Head and Neck | 1.03 | 1.31* | 3.05 | 1.89*# | 3.43 | 1.97* |
| Esophagus | -0.04 | 0.98 | 0.88 | 1.52* | 0.59 | 1.33 |
| Stomach | 0.47 | 1.18* | 0.90 | 1.35* | 1.25 | 1.49 |
| Colon Excluding Rectum | 1.35 | 1.11* | 1.56 | 1.13* | 2.58 | 1.22 |
| Rectum and Rectosigmoid | -0.55 | 0.87* | -1.24 | 0.70* | 0.45 | 1.12 |
| Anus, Anal Canal and Anorectum | 0.30 | 1.77* | 0.67 | 2.51* | 0.14 | 1.29 |
| Liver, Gallbladder and Biliary | 0.34 | 1.12 | 0.55 | 1.17 | 2.28 | 1.63* |
| Pancreas | -0.33 | 0.92 | -0.75 | 0.84 | 1.21 | 1.24 |
| Lung and Mediastinum | 6.64 | 1.32* | 6.90 | 1.31* | 5.81 | 1.26* |
| Bone and Joint | 0.19 | 2.43* | 0.37 | 3.64* | 0.33 | 3.24 |
| Soft Tissue | 0.13 | 1.19 | 0.51 | 1.65* | 1.03 | 2.18* |
| Melanoma | 1.93 | 1.37* | 1.24 | 1.20* | -0.22 | 0.97 |
| Breast | -2.78 | 0.83* | 2.35 | 1.13*# | 2.58 | 1.15# |
| Female Breast | -2.83 | 0.82* | 2.08 | 1.12*# | 2.32 | 1.13# |
| Male Breast | 0.05 | 1.28 | 0.27 | 2.41* | 0.27 | 2.26 |
| Gynecologic | -0.57 | 0.91 | 0.20 | 1.03 | -1.02 | 0.85 |
| Prostate | -1.87 | 0.92* | -2.31 | 0.91* | -2.07 | 0.91 |
| Testes | 0.01 | 1.03 | -0.06 | 0.72 | -0.21 | 0.00 |
| Penis | 0.01 | 1.10 | 0.05 | 1.36 | 0.01 | 1.05 |
| Bladder | 0.97 | 1.12* | 3.61 | 1.41*# | 8.04 | 1.84*# |
| Kidney and Renal Pelvis | 2.37 | 1.61* | 0.71 | 1.16# | 2.03 | 1.42* |
| Brain | 0.27 | 1.19 | -0.21 | 0.86 | -0.89 | 0.41 |
| Thyroid | 2.14 | 2.45* | 0.75 | 1.45*# | 1.43 | 1.76* |
| Hodgkin Lymphoma | 2.17 | 7.06* | 2.53 | 7.97* | 0.90 | 3.47* |
| Myeloma | 0.10 | 1.05 | 0.17 | 1.08 | 0.57 | 1.23 |
| Leukemia | 6.31 | 2.55* | 3.93 | 1.87*# | 1.60 | 1.33# |
| Mesothelioma | 0.08 | 1.20 | 0.18 | 1.42 | 0.49 | 2.07 |
| Kaposi Sarcoma | 1.71 | 11.50* | 0.19 | 2.37# | -0.11 | 0.00# |
| Miscellaneous | 1.04 | 1.32* | 1.66 | 1.50* | 1.47 | 1.45 |

*P<0.05 Observed vs. Expected relative to endemic population rate

#P<0.05 Compared to 2-119 months and relative to each endemic population rate
